# Non-Invasive Preimplantation Genetic Testing for Aneuploidy and the Mystery of Genetic Material: A Review Article

**DOI:** 10.3390/ijms23073568

**Published:** 2022-03-25

**Authors:** Maja Tomic, Eda Vrtacnik Bokal, Martin Stimpfel

**Affiliations:** 1 Department of Human Reproduction, Division of Obstetrics and Gynecology, University Medical Center Ljubljana, 1000 Ljubljana, Slovenia; may.tomic@gmail.com (M.T.); eda.bokal@guest.arnes.si (E.V.B.); 2 Faculty of Medicine, University of Ljubljana, 1000 Ljubljana, Slovenia

**Keywords:** aneuploidy, blastocoel, cell free DNA, embryo biopsy, preimplantation genetic testing, spent embryo culture media, trophectoderm biopsy

## Abstract

This review focuses on recent findings in the preimplantation genetic testing (PGT) of embryos. Different preimplantation genetic tests are presented along with different genetic materials and their analysis. Original material concerning preimplantation genetic testing for aneuploidy (PGT-A) was sourced by searching the PubMed and ScienceDirect databases in October and November 2021. The searches comprised keywords such as ‘preimplantation’, ‘cfDNA’; ‘miRNA’, ‘PGT-A’, ‘niPGT-A’, ‘aneuploidy’, ‘mosaicism’, ‘blastocyst biopsy’, ‘blastocentesis’, ‘blastocoel fluid’, ‘NGS’, ‘FISH’, and ‘aCGH’. Non-invasive PGT-A (niPGT-A) is a novel approach to the genetic analysis of embryos. The premise is that the genetic material in the spent embryo culture media (SECM) corresponds to the genetic material in the embryo cells. The limitations of niPGT-A are a lower quantity and lesser quality of the cell-free genetic material, and its unknown origin. The concordance rate varies when compared to invasive PGT-A. Some authors have also hypothesized that mosaicism and aneuploid cells are preferentially excluded from the embryo during early development. Cell-free genetic material is readily available in the spent embryo culture media, which provides an easier, more economic, and safer extraction of genetic material for analysis. The sampling of the SECM and DNA extraction and amplification must be optimized. The origin of the cell-free media, the percentage of apoptotic events, and the levels of DNA contamination are currently unknown; these topics need to be further investigated.

## 1. Introduction

As parental age soars, so does infertility. Infertility is clinically defined as a disease of the reproductive system, characterized by failure to achieve a clinical pregnancy after at least 12 months of regular unprotected sexual intercourse [1]. The median prevalence of infertility is estimated at approximately 9%, with the percentage being higher in more developed countries [2].

Counseling, pharmacotherapy, surgery, and assisted reproductive technology (ART) are all part of the treatment of infertility [2]. In the last decade, there has been a shift towards elective single embryo transfer (eSEP). Elective single embryo transfer is a method where the embryo with the best chance for survival is selected for implantation. With the selection of a single embryo, eSEP minimizes the complications that arise from a multiple pregnancy [3]. While the eSEP method lowers the number of viable pregnancies in comparison with double embryo transfer in a fresh cycle of in vitro fertilization (IVF), an additional frozen eSEP cycle minimizes this difference [4].

The best embryo is frequently selected based on morphological assessment, as this is a cost-effective, rapid, and non-invasive technique [3,5]. While it is widely used, this technique does not account for the possibility of genetic abnormalities, since there is no correlation between morphological traits and genetic material [6]. Chromosomal aneuploidy can occur in 20–80% of human embryos [7].

A conclusion that has been drawn in multiple studies is that aneuploidy can cause unsuccessful implantation or an early miscarriage [3,8,9]. Therefore, there is a growing demand for routine testing and the selection of euploid embryos for implantation, as this will help to achieve better implantation rates of the embryos, a smaller rate of early miscarriages, and, consequently, a higher rate of live births [3]. The technique that would allow for such testing is called preimplantation genetic testing for aneuploidies (PGT-A).

Currently, the most widely used form of PGT-A is an invasive biopsy of the trophectoderm (TE), followed by next generation sequencing (NGS) [3]. The invasive biopsy of TE improves embryo selection, implantation and pregnancy rates, and the number of live births [10]. It also requires a team of highly trained embryologists, since the procedure is very complex and invasive [11]. Because the method of genetic testing varies, the procedure is not standardized and the results between different centers vary substantially. One of the bigger problems is mosaicism, a phenomenon where there are several cell lines within a single embryo. This creates a probability that the biopsied cells will not hold genetic material that represents the embryo’s genetic material [10].

With any method of invasive testing, there are ethical dilemmas. In some countries, embryo biopsy is not allowed, which also presents legal dilemmas [11]. Because of the reasons stated, the scientific community is leaning towards developing and implementing a non-invasive PGT-A (niPGT-A) or a minimally invasive PGT-A (miPGT-A). Cell-free DNA (cfDNA) is a fraction of the genetic material found in the spent embryo culture media (SECM) that can be readily isolated and sequenced, producing a result that has been shown to be highly concordant with invasive PGT-A in many different studies [12]. When performing niPGT-A, a laser is not required and neither is the highly trained workforce, which makes the method more cost-effective. The most attractive feature is, of course, the prospect of non-invasiveness [13]. However, mosaicism is prevalent even in niPGT-A.

## 2. Emerging Biological Markers in SECM

### 2.1. Extracellular Vesicles

Extracellular vesicles (EVs) are biological nanoparticles consisting of a lipid bilayer that enables them to cross cell membranes freely. There is a wide variety of cargos encapsulated inside the EV that can be readily internalized by recipient cells. EVs are classified according to their biogenesis, and their origin can usually be predicted based on their size and membrane composition [3]. There are three main classes: exosomes, microvesicles, and apoptotic bodies [14,15] (Table 1).

The EVs are released at all stages of preimplantation development, and can readily cross the zona pellucida, which enables the embryos to communicate with their surroundings [5,15]. Exosomes, in the manner described earlier, act as paracrine factors that help regulate blastocyst development in a shared medium in an in vitro setting [15].

Palliger et al. published a study in 2016 describing how EV concentrations in their study were significantly higher in human non-competent embryos than in competent embryos [16]. On the other hand, another study on human embryos showed no differences in EV concentrations among embryos with different developmental competence. In the same study, however, they evaluated the diameter of EVs in comparison with embryo quality and discovered that the embryos with the best quality on day three had a larger EV diameter compared to poor-quality embryos. The authors of the said study also performed aCGH on developmentally arrested human embryos as well as on media culture samples and discovered a lower rate of chromosome abnormalities in the arrested embryos, rather than in their corresponding EVs, suggesting that the self-correcting mechanisms of aneuploid embryos could explain this [17]. As Palliger et al. predicted in their study, the implementation of the measurement of EV concentration under standardized protocols could offer a simple, non-invasive, and inexpensive test, which could ultimately contribute to choosing the most prosperous embryo with the highest chance of implantation in a receptive endometrium [16].

### 2.2. MicroRNA

MicroRNAs (miRNAs) are short non-coding RNA molecules up to 22 nucleotides long that are stable and readily recognizable, which could make them an attractive new biological marker [3]. MiRNA molecules are derived from single-stranded RNA molecules that form double-stranded hairpin structures. An exonuclease named Dicer then clips and processes these hairpin structures. The formation of miRNA is complex and involves both nuclear and cytoplasmic phases [18]. There appear to be more than 135 different miRNA molecules present in SECM at the blastocyst stage, where they might play an important role in the growth and development of the embryo [5,19,20]. Both the oocytes and spermatozoa contribute their share of miRNA molecules [3]. The profile of miRNA molecules in the SECM differs greatly. For example, the miRNA profile in SECM is seems to be different if either of the parents has known infertility issues [21]. The miRNA profile also appears to be different in the case of euploid and aneuploid embryos. Certain miRNA molecules could be more prevalent in male blastocysts, which could indicate a certain level of sexual differentiation at the blastocyst stage [20].

In 2014, Rosenbluth et al. discovered a higher concentration of an miRNA named miR-191 in the SECM of aneuploid embryos, and speculated that miR-191 could be used as a biological marker for embryo ploidy. They also discovered a miRNA molecule named miR-372, which they speculated could, in high concentrations, predicted IVF failure; however, it was not found to be relevant in the determination of the embryo ploidy. In their study, pregnancy outcomes and miRNA profiles were correlated only when intracytoplasmic sperm injection (ICSI) was used, which could limit the use of miRNA as a biological marker for pregnancy outcomes [22]. Fang et al. also concluded that certain miRNA molecules (namely, has-miR-26b-5p and has-miR-21b-5p) could serve as potential biomarkers for reproductive outcomes [23].

A 2016 study by Capalbo et al. was among the first to comprehensively characterize the miRNA population secreted from human blastocysts. They discovered many secreted miRNA molecules (14 out of 59 detected miRNA types) that seemed to be predicted to be involved in endometrial cell growth and proliferation, which could point to a potential communication system between the embryo and endometrium at the implantation site [24].

These authors cumulatively agree that miRNA could serve as a good non-invasive marker of the quality of the preimplantation embryo, as it is stable, easily accessible, and easily detected. More research is needed to standardize the tests and to better understand the role of these molecules [22,23,24].

## 3. Genetic Material

### 3.1. Cell-Free DNA in Spent Embryo Culture Media

Cell-free DNA in SECM was discovered in 2013 [25]; however, numerous authors have since agreed that not all cfDNA is embryonic in origin [25,26,27]. In 2018, Vera-Rodriguez et al. led a study where they aimed to interpret cfDNA and determine its level of contamination with maternal genetic material. The authors compared the results with PGT-A for better interpretation. They concluded that the cfDNA was not relevant to the ploidy of the embryo. They also reported contamination with maternal genetic material in an estimated 86–94% of cases. The same problem with maternal contamination has been highlighted by several authors and remains one of the relevant obstacles in the interpretation of cfDNA [7,28,29]. When using a method that searches for single-nucleotide polymorphisms (SNP), contamination with maternal genetic material becomes more prominent. In one study, this method also revealed that the percentage of embryonic DNA in SECM varied between 0% and 100%, which suggests the embryonic genome may not be uniformly represented in the SECM of all embryos [7].

### 3.2. Cell-Free DNA in the Blastocoel

On the fourth day of blastocyst development, cells begin to differentiate into trophectoderm (TE) cells and the inner cell mass (ICM) [5]. During cavitation, a cavity named the blastocoel is formed inside the blastocyst. It is filled with blastocoel fluid (BF) [30]. CfDNA in the BF (BF-cfDNA) was first detected in 2013 [31].

BF can be readily aspirated from the blastocyst using a procedure called blastocentesis, which could become an alternate for blastocyst biopsy as a miPGT-A method [32,33,34]. Another way for the BF to exit the blastocoel can be during PGT-A biopsy or during vitrification. In the first scenario, a laser cuts between TE cells extruding through zona pellucida. In the later scenario, the BF is removed to prevent ice crystal formation before cryopreservation, which is again achieved with a laser pulse between TE cells [35]. After the BF is expelled from the embryo, the blastocoel usually collapses upon itself [33,35]. The BF conditioned media is therefore a mixture of BF that leaks into the culture media and is not to be mistaken with SECM, which is embryo-conditioned growth media [35].

Generally, only a very small amount of approximately 0.01 µL of BF can be isolated [27]. Considering this, the concentration of cfDNA available in the BF is minimal but nevertheless is sufficient for amplification in the majority of cases [36]. In their 2018 study, Capalbo et al. reported that only a portion of BF samples (34.8%) could generate a signal that could be used for embryo karyotyping. They attributed this result to the blastocentesis being performed on freshblastocysts [28].

One of the biggest setbacks in implementing miPGT-A methods with blastocentesis could be a low concordance rate between BF-cfDNA and the TE biopsy. Capalbo et al. reported a 37.5% concordance rate [28], while Tšuiko et al. reported a 40.0% concordance rate between the BF and TE biopsies [34]. Capalbo et al. speculated that this could be due to the unknown origin of cfDNA [28]. Many studies have come to the same conclusion and have suggested that the genetic material could be due to apoptosis, a defective chromosome segregation process, or the selective degeneration of abnormal cells in mosaic embryos [28,30,34,37]. One study described a correlation between the ploidy status of an embryo and the cfDNA in blastocoel fluid-conditioned media, wherein they reported higher concentrations of cfDNA with euploid embryos than with aneuploid embryos [33].

### 3.3. Mosaicism

Mosaicism is a phenomenon where there are multiple cell lines within one embryo [10]. Most commonly mosaicism is classified into three groups: chaotic mosaicism, aneuploidy mosaicism, and diploid-polyploid mosaicism [38,39]. Delhanty et al. published a paper in 1997 and coined the term chaotic mosaicism to describe a severe pattern of irregularity and imbalances for multiple chromosomes which differed between cells [38,40,41]. Aneuploid mosaicism is a phenomena where normal cell lines and distinct aneuploidy complements coexist in the same embryo [38], while diploid-aneuploid mosaicism describes the type of mosaicism that involves a trisomy and/or monosomy for only one of the chromosomes analyzed [38,42].

There have been many theories trying to explain the mechanism of mosaicism formation. In one of the early studies, anaphase lagging has been highlighted as one of the more prevalent mechanisms [43]. Other more prevalent mechanisms include non-disjunction [43,44,45] and endoreplication [44,46].

In the early pre-implantation stage of embryo development, mosaicism is quite prevalent; it appears in an estimated 10–30% of embryos [39], or by some sources in even as much as 50% of all embryos [47]. However, the prevalence of mosaicism seems to plummet during the later progression of pregnancy [48]. True fetal mosaicism is, by some estimates, prevalent in only approximately 0.4% of fetuses, and mosaicism in live births has been estimated at <0.2% [49]. Maternal age does not seem to affect mosaicism [50].

In a small study, conducted on 11 blastocysts, Orvieto et al. concluded that the embryo has the ability of what they called “self-correction”. This so-called self-correction is the ability of the embryo to expel abnormal cells and/or fragments [47]. With this idea, the authors concurred with Gleicher and Barad, whose shared opinion was that any diagnostic measure at the blastocyst stage is to be questioned, as testing at that stage usually does not take into account the self-correction mechanisms of the embryo [47,51]. Some researchers have recently proposed the possibility of transferring some types of mosaic embryos [50,52,53,54]. This proposal has since received mixed reviews. If adhering to the proposed guidelines, one must be careful to deprioritize mosaic transfers according to the chromosome types that are compatible with live births in pure aneuploid form (such as X; Y; and trisomy 13, 18, or 21) [54,55,56]. While the rate of miscarriages when using PGT-A is low, more than 50% of all miscarried embryos were discovered to be mosaic, which led Munné et al. to speculate that the miscarriage rate could be even lower if only euploid embryos were transferred [52].

### 3.4. Parental Age and the Quality of the Genetic Material

The topic of parental age during IVF treatment has been controversial As international guidelines are scarce, this topic is regulated individually by each country.

#### 3.4.1. Contribution of Maternal Age

Ubaldi et al. conducted a multicenter, longitudinal, observational study in 2017 where they observed laboratory and clinical outcomes in pre-implantation genetic testing for aneuploidy in women between the ages of 44 and 47. The authors defined the primary outcome as live birth per started treatment. The secondary outcomes were biochemical pregnancy loss, miscarriage, and chromosomally abnormal pregnancy rates. The primary outcome was achieved in 12 out of 150 cases, or in 8% of the cases, in the maternal age interval of 44–47. If the outcomes were grouped based on the maternal age, 11 out of 12 live births were recorded in the group with a maternal age of 44.0–44.9, one birth was reported in the group where the maternal age was 45.0–45.9, and there were no live births beyond the age of 46.0 [57].

In a paper published by Munné et al. in 2016, the correlation between embryo diagnosis and maternal age is apparent. The embryo diagnoses were as follows: euploid, aneuploid, mosaic and aneuploid, or mosaic aneuploid/euploid. The authors discovered that as maternal age progresses, so does the percentage of aneuploid embryos and mosaic aneuploid embryos, while the percentage of euploid embryos declines. The percentage of mosaic aneuploid/euploid embryos progresses at first and then declines [52].

Sawarkar et al. conducted a multicenter study in 2021 that evaluated chromosome abnormality rates in embryos of patients in the same age group. The authors concluded that maternal age is only a gross predictor of chromosomal abnormalities. They found patients in each age group who had all the embryos in their cohort that were either normal or abnormal. In line with previous studies, the trend towards increased abnormality was observed as the patients’ age progressed. In the 41–45 age group, only 20% of embryos were euploid. In addition to this, only 6% of patients in the 38–40 age group had a chance of having 75–100% normal embryos, compared to 40% of patients in the below 35 age group [58].

In 2013 Harton et al. conducted a study and concluded the euploid embryo implantation rate is constant and unrelated to maternal age. The results were statistically significant for patients aged 42 or younger [8].

#### 3.4.2. Contribution of Paternal Age

At least 30% of all couple infertility is paternal in origin [59]. In an older study, which followed 5121 California women between the years 1990 and 1991 they discovered that, if paternal age was greater than 35 years, women were 1.26 times more likely to miscarry than women whose partners were younger than 35 years [60]. In another study, where intrauterine insemination was used in more than 17,000 cycles, the partners of men older than 35 years had a miscarriage rate of 32.4%, while the partners of men younger than 35 years had a miscarriage rate of 13.7%. In the same study, pregnancy rate decreased from 12.3 % (before 30 years of age) to 9.3 % (after 45 years of age) [61].

Since spermatozoa undergo mitotic divisions throughout a man’s life, the risk for DNA fragmentation and deleterious point mutations rises with age. De novo autosomal dominant mutations are more prominent, while autosomal aneuploidies do not increase with paternal age. There is also an increase in various diseases, including X-linked diseases (such as hemophilia A and B, Hunter syndrome, and Duchenne muscular dystrophy); pediatric cancers (such as leukemia, non-Hodgkin’s lymphoma, pediatric central nervous system tumors, and breast cancer); congenital anomalies (such as diaphragmatic hernia, pulmonary stenosis, and cleft lip); psychiatric disorders (such as schizophrenia and bipolar disorder); and neurological and developmental disorders (such as autism, which is six times more likely if paternal age is 40–49 years, and lower scores on cognitive tests) [59,62].

One method of treating male infertility is with IVF, either with or without ICSI. ICSI is an invasive method, with the potential to cause epigenetic changes in early embryogenesis. It also enables the transfer of lower-quality sperm, which could affect the health and fertility of the offspring. ICSI has been in use since 1991, and scientists are now able to study and discover the potential effects in young adults who were conceived this way [63]. Different researchers have discovered that young male adults conceived with ICSI have lower-quality sperm, potentially higher levels of FSH, and lower or normal levels of inhibin B in comparison with other young men [59,63]. Whether this is in part hereditary cannot yet be excluded [59].

## 4. Preimplantation Genetic Testing

### 4.1. Invasive Preimplantation Genetic Testing

The development of PGT-A has increased pregnancy rates and reduced miscarriage rates especially in older women. This did not seem to improve the cumulative pregnancy rate in older women [57]. Because the process is invasive, some papers have suggested this may reduce embryo quality and could potentially cause a failure to implant [50,64].

Polar body (PB) biopsy was one of the first types of pre-implantation genetic testing (PGT) [65]. During meiosis of the oocyte, two PBs are produced: one during ovulation and the other after oocyte fertilization. Because PBs are not an integral part of the embryo, this technique is considered to be less invasive [30]. Despite this consideration, Levin et al., in their 2012 study, reported a higher percentage of fragmentation of the embryo’s DNA and a lower number of blastomere cells on the second and third day. This shows that even PB biopsy may have an effect on the embryo to some extent [66]. The main disadvantage of this method is that it only provides information about the maternal genetic material [30].

A method that was frequently used in the past was blastomere biopsy. In this method, a biopsy of 1–2 blastomeres was performed on the third day after fertilization, when the embryo typically has 6–10 cells [30]. Because the rate of chromosomal mosaicism is at its highest at that time, this can lead to a mistake in the execution and interpretation of PGT-A [67]. There have also been reports of changed morphokinetic properties of the embryo, which could lower the embryo’s implantation potential [68]. In 2009, Yu et al. conducted a study on mice. They found poorer memory function and hypomyelination in mice that had undergone a blastomere biopsy during embryological development [69]. In another study, mice exhibited changes in the structure and function of the adrenal glands, and a poorer glucocorticoid response to cold [70].

The implementation of TE biopsy is the latest evolution of PGT-A. Currently in use is a method referred to as PGT-A 2.0, which consists of a TE biopsy and further sequencing of the genetic material with NGS [10]. Some sources call the technique PGT-A 3.0 [50]. The TE biopsy is performed on the fifth to seventh day after fertilization [9]. Choosing the most genetically prosperous embryo is the main advantage of PGT-A 2.0, which in turn increases the chances of a healthy newborn. The disadvantages of PGT-A 2.0 lie in the method itself, as it is invasive and elaborate. Because of this, highly trained embryologists and a state-of-the-art laboratory that is capable of growing and freezing embryos are needed [10].

A TE biopsy is a good predictor of the chromosomal status of the entire embryo in euploid embryos or whole chromosomal aneuploidy. Its predictive value, however, is reduced in the case of embryonic mosaicism [53]. A study conducted in 2019 by Zhang et al. described a connection between TE biopsy and an increased probability of pre-eclampsia in pregnant women. The risk of pre-eclampsia was three-fold greater than in the population of pregnant women that used IVF but not PGT-A [71].

### 4.2. Preimplantation Genetic Testing of Cell-Free Genetic Material

Currently, researchers are developing new methods and technologies for minimally invasive preimplantation genetic testing (miPGT-A) and niPGT-A. These methods test the genetic material in BF, SECM, or both BF and SECM [13]. The obvious advantage of these methods is the non-invasiveness or minimal invasiveness of the technique. Other advantages include cost efficiency, time efficiency, and a lesser need for extensively trained staff [11,13].

#### 4.2.1. niPGT-A and miPGT-A Represent a Step Forward in the Artificial Reproductive Technology

In 2020, Rubio et al. published a large multicenter prospective study, showing concordance in the sensitivity and specificity of PGT-A and niPGT-A. To date, this is one of the largest studies of its kind, as the authors tested 1301 blastocysts. Since this was a multicenter study, the concordance rate between PGT-A and niPGT-A ranged from 76.5% to 91.3% in terms of their sensitivity, and from 64.7% to 93.3% in terms of their specificity. They concluded that niPGT-A is a reliable tool, as long as the protocols and algorithms for testing are determined in advance [12].

Rubio et al. tested 81 embryos that were donated to science. They tested cfDNA from the SECM and DNA from TE and ICM biopsies, and their results showed good concordance rates. They reasoned that cfDNA material must be contributed both by TE and ICM cells [12]. Two years before this research, Gleicher and Barad posed doubts about that same preposition, hypothesizing that SECM should contain cfDNA material contributed to predominantly by TE cells, because they are the only cells that touch the SECM [51].

Rubio et al. concluded that different ovarian stimulation protocols, culture conditions, and the quality of the embryo do not affect the accuracy of niPGT-A, and neither does a smaller drop of culture medium [12]. In fact, a smaller volume of culture medium seems to promote the growth and development of the embryo [72]. Different authors have proposed that the prosperous growth of the embryo in a smaller culture medium volume is the result of autocrine and paracrine growth factors that the embryo releases in the time before implantation [3,72].

#### 4.2.2. Unanswered Questions Are Halting Implementation

A 2017 study on 32 embryos proved that cfDNA in BF is positively correlated with embryo morphology [37], which is the exact opposite result to that of other studies in which no correlation between the two was found [6,73]. A year later, Tšuiko et al. isolated genetic material from the BF, ICM, and TE of 16 frozen blastocysts and discovered that the genetic material could be reliably amplified and sequenced by NGS only from the TE and ICM biopsies. The results of the TE and ICM biopsies were concordant. Meanwhile, the cfDNA from BF could not always be amplified and the results did not necessarily reflect the results of the TE or ICM biopsies [34].

In some of these studies, the blastocyst was frozen beforehand, which could promote the lysis of cells and, in turn, amplify the quantity of cfDNA in the BF [34,74]. If the embryos are not frozen, the cfDNA concentration could be much lower, as Capalbo et al. concluded in their study where they successfully generated a signal that could be used for embryo karyotyping purposes in only 34.8% of embryos [28]. Considering this, Tšuiko et al. could have had even less favorable results if the embryos had not undergone the freezing process.

Hanson et al. voiced their doubts about the efficiency of niPGT-A in their 2021 study. In their research, the amplification rate of DNA was 100% if it was isolated via PGT-A methods, and 62.7% if it was isolated via niPGT-A. Discrepancies between the niPGT-A and TE biopsy results after sequencing were found in 40.4% of the cases. Furthermore, three embryos that were classified as aneuploid by niPGT-A biopsy results successfully developed into three healthy newborns [75]. The authors attributed these discrepancies to differences in study design. While some authors, Rubio et al. for example, used smaller droplets of medium [12,72], Hanson et al. used the medium as per the manufacturer’s instructions [75]. Hanson et al. also pointed out that in those studies, the embryos required prolonged exposure to the culture medium for the DNA amplification to be successful (on day 6/7 rather than on day 5, as described above), delaying the biopsy and vitrification of the embryo. They deemed this irresponsible, as it would ultimately harm the embryos. They suggested that studies in advance become more applicable to clinical settings [75].

Many authors have raised concerns about the origin of the cfDNA in SECM and BF. They argue the source of cfDNA must be found before the implementation of miPGT-A and niPGT-A in clinical practice [12,27,76]. They estimate that only about 8% of cfDNA is embryonic in origin [7,27].

Some have hypothesized that cfDNA correlates with apoptotic events [47,77]. Other studies, however, have found that there is no statistically important difference between the concentration of cfDNA fragments in SECM, no matter the quality of the blastocyst [14] or the ploidy of the embryo [7].

Gombos et al. published a retrospective study in 2021 in which they sampled SECM on day 3. The pivotal point of their research was setting the endpoint of their analysis as the pregnancy outcome. This enabled them to compare the SECM of embryos that resulted in successful pregnancies and those that resulted in abortion after successful implantation. The cfDNA in the SECM of the aborted embryos was found in higher copy numbers, while the low embryonic cfDNA in SECM was consistent with a healthy pregnancy and live birth [78]. While the causation of the abortions was unknown, this indicates a correlation between cfDNA concentration and pregnancy outcomes.

Huang et al. proposed that SECM must contain more cfDNA from euploid cells rather than the aneuploid cells, otherwise niPGT-A would not be as successful [76]. Orvieto et al. questioned this proposal in their research. While their sample size was small (11 blastocysts), the results showed that 63.3% of blastocysts expelled cell debris with additional chromosomal rearrangements. Of nine euploid blastocysts, five (55.5%) expelled aneuploidy debris. The authors concluded that these results were the opposite of what Huang et al. proposed [47].

Part of the origin of cell-free genetic material could be from EV and miRNA secretion [3,13]. A portion of cfDNA could also be a product of maternal cell contamination [13,27,76]. In their study, Vera-Rodriguez et al. found cfDNA with only an XX karyotype in the SECM of XY embryos in 30.4% of the cases, and cfDNA with both an XX and XY karyotype in an additional 30.4% of the cases [7]. In their research, Chen et al. found that maternal contamination could mainly come from cumulus cells and the PBs [79]. Multiple authors have proposed that cfDNA could be a byproduct of the self-repairing mechanisms of the embryo, in which case cfDNA would be a residue of excluded cells [47,51,80].

## 5. Discussion

Preimplantation genetic testing for aneuploidy is an increasingly relevant tool in artificial reproductive technology. Currently, the most commonly used tool is PGT-A 2.0, which has the main disadvantage of being an invasive method. This can result in unpredictable and largely unknown side effects that are only now being discovered [29,68,69,70,71]. Because of this, niPGT-A and miPGT-A are currently being developed and standardized for future clinical practice.

While the majority of the scientific community agrees that niPGT-A, or at least miPGT-A, should be implemented in clinical practice, there are many doubts and concerns that must be addressed beforehand. One concern, for example, is the origin of cfDNA, which is not yet known, and neither is the level of contamination [51,75]. Supporters of the niPGT-A and miPGT-A techniques argue that the methods have been thoroughly researched and that the results have been concordant with PGT 2.0 [12,72].

Another critique aims at all preimplantation genetic testing for aneuploidy. Some believe that determining the ploidy and quality of the embryo at the blastocyst stage is inadequate either way, as many repair mechanisms will start working at a later phase of development [47,51]. This would also explain the high prevalence of mosaicism in the embryo but a rather low prevalence of mosaicism in the population [47,49,80]. On the other hand, multiple studies have continuously reached statistically significant results showing that preimplantation genetic screening for aneuploidy and mosaicism allows for more desirable IVF results [3,8,9].

The genetic material in the BF and SECM is not limited to cfDNA. Researchers have found a whole array of miRNA and EVs carrying genetic material. While these molecules do not carry the chromosomal information of the embryo, their representation and concentration in the medium can predict ploidy, implantation rate, and certain diseases. At the same time, they act as autocrine and paracrine factors, and may act as gross predictors of embryo quality [3,20,21,22].

## 6. Conclusions

While less invasive preimplantation genetic testing methods are very amiable, further research is needed to determine the origin of cfDNA. As certain liberties have been taken in the protocols of some studies [12], all protocols must be standardized and unified before niPGT-A and miPGT-A can be implemented in clinical practice. MiRNA and EVs are promising biological markers that could serve as a cheap, non-invasive prediction tool beyond preimplantation genetic testing for aneuploidy; however, further studies are needed to determine the exact profiles and concentrations.

## Figures and Tables

**Table 1:** Cargo and size differences between different types of EVs [3,14,15].

Type of EV	Size of the Nanoparticle	Typical Cargo
Exosomes	30–120 nm	mRNA, non-coding RNA (including miRNA), cytoplasmic and membrane proteins
Microvesicles	50–1000 nm	mRNA, non-coding RNA (including miRNA), cytoplasmic and membrane proteins
Apoptotic bodies	500–2000 nm	Nuclear fractions, cell organelles
